# Changes in urinary albumin levels with dotinurad oral administration in hyperuricemic patients with microalbuminuria: a post hoc analysis

**DOI:** 10.1007/s10157-025-02750-4

**Published:** 2025-08-21

**Authors:** Toshinari Takahashi, Takanobu Beppu, Tatsuo Hosoya, Naoto Yokota

**Affiliations:** 1 Medical Affairs Department, Mochida Pharmaceutical Co., Ltd, 7 Yotsuya 1-chome, Shinjuku-ku, Tokyo 160-8515 Japan; 2 Medical Affairs Department, Fuji Yakuhin Co., Ltd, 9 F Kanda Square Building, 2-2-1 Kandanishiki-cho, Chiyoda City, Tokyo 101-8189 Japan; 3 Jikei University School of Medicine, 3-25-8 Nishi-Shimbashi, Minato City, Tokyo 105-8461 Japan; 4 Yokota Naika, 642-1 Komuta Hanagashimacho, Miyazaki City, Miyazaki 880-0036 Japan

**Keywords:** Albuminuria, Dotinurad, Selective urate reabsorption inhibitor, SURI, URAT1, Urate transporter 1

## Abstract

**Background:**

Albuminuria is an important indicator of kidney damage. It increases the risk of adverse outcomes such as end-stage renal failure and cardiovascular events. Dotinurad, a drug newly developed in Japan, selectively inhibits renal urate transporter 1. This post hoc analysis of a Japanese, multicenter, phase 3 clinical trial (ClinicalTrials.gov identifier, NCT03006445) of dotinurad in patients with hyperuricemia investigated the effects on urinary albumin levels among a subset of patients with microalbuminuria.

**Methods:**

The study endpoint was the change in urine albumin-to-creatinine ratio (UACR) from baseline at Weeks 34 and 58. Subgroup analyses by patient background and clinical characteristics assessed potential correlations with the change in UACR.

**Results:**

Data from 39 patients were analyzed. All were male (mean age, 57.5 years). At Week 34, the geometric mean of the change in UACR was −43.5% (95% confidence interval [CI] −55.3%, −28.6%; linear mixed effects model, p < 0.05). At Week 58, the geometric mean of the change was −41.5% (95% CI −73.5%, 29.1%; linear mixed effects model, p < 0.05). There were no significant correlations between the rate of change in UACR and background characteristics such as body mass index or complicating diabetes. Systolic blood pressure level was significantly correlated with the rate of change in UACR at Week 34.

**Conclusion:**

A significant decrease in UACR following dotinurad administration was observed. Additional RCTs with appropriate control groups are needed to further confirm these findings.

**Supplementary Information:**

The online version contains supplementary material available at 10.1007/s10157-025-02750-4.

## Introduction

In Japan, chronic kidney disease (CKD) is an important public health problem, with one in eight adults reported to have CKD [1]. The risk of outcomes such as end-stage renal failure requiring dialysis, cardiovascular events, and death increase according to the degree of albuminuria. Microalbuminuria (urine albumin-to-creatinine ratio [UACR] 30–299 mg/g) is thought to be an early sign of kidney damage and is associated with CKD progression [2, 3]. Treatments that decrease albuminuria, such as sodium-glucose cotransporter-2 inhibitors, mineralocorticoid receptor antagonists, angiotensin-converting enzyme inhibitors, and angiotensin II receptor blockers, may reduce the risk of progression to kidney failure [4–6]. Studies in patients with type 2 diabetes have highlighted the importance of the early detection and management of microalbuminuria in preventing progression to kidney disease [7, 8]. A recent review also emphasizes the importance of albuminuria as a risk factor for cardiovascular disease and thus of its surveillance and management [9].

Hyperuricemia has been recently associated with lifestyle-related diseases—the so-called metabolic syndrome [10, 11]. Reported etiologies of CKD include hypertension, diabetes, age, family history, metabolic syndrome, and smoking [12–14]. Patients presenting with albuminuria have a poorer prognosis than those who do not, and the risks of cardiovascular disease events and cardiovascular death are reported to increase with increasing albuminuria [12, 15–17].

Hyperuricemia is generally classified based on uric acid overproduction or underexcretion. Xanthine oxidase inhibitors, such as allopurinol, febuxostat, and topiroxostat are used to inhibit the production of uric acid, and uricosuric drugs, such as probenecid and benzbromarone, are used to treat underexcretion [18]. Interestingly, topiroxostat and febuxostat have also been reported to reduce levels of urinary albumin [19, 20].

Dotinurad is a novel urate reabsorption inhibitor that selectively inhibits urate transporter 1 (URAT1) and has potent uric acid-lowering activity in the blood [21]. A clinical trial investigating long-term dotinurad treatment (up to 58 weeks) in patients with hyperuricemia demonstrated that 100% of patients achieved a reduction in serum uric acid to ≤ 6.0 mg/dL [22]. In contrast with conventional uricosuric drugs such as benzbromarone and probenecid that may exhibit hepatotoxicity or drug–drug interactions, dotinurad may not have such adverse effects because it is highly selective for URAT1 and also has a structural benefit as it does not contain the features thought to be responsible for the toxic effects of benzbromarone [18, 23, 24].

While the evidence for the serum uric acid-lowering effect of dotinurad is sufficiently comparable to conventional drugs, there is little evidence for its effects on urinary albumin excretion. This post hoc analysis of a phase 3 clinical trial of oral administration of dotinurad to patients with hyperuricemia investigated the effects on urinary albumin levels at 34 and 58 weeks in a subset of patients with microalbuminuria.

## Patients and methods

### Data set

Data collected in the phase 3, multicenter, open-label, dose-escalation study of dotinurad treatment for 34 or 58 weeks at 26 clinical institutions in Japan (ClinicalTrials.gov identifier NCT03006445) were used in this post hoc analysis [22, 25]. The clinical trial was based on International Council for Harmonisation E1 guidelines and required 100 patients to receive the maintenance dose for at least 1 year. The first 120 of the planned 330 patients would be allocated to the 58-week group, and the remaining 210 patients would be allocated to the 34-week group in anticipation of drop-outs and discontinuations. The final number of patients allocated was 299 in the 34-week group and 108 in the 58-week group.

**Fig. 1:**
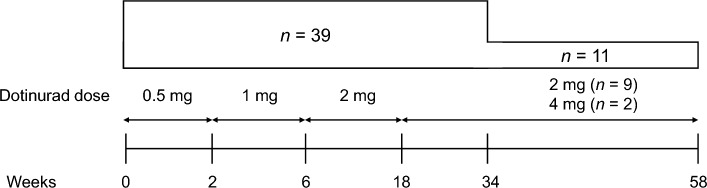
Study design

Dotinurad was initiated at 0.5 mg/day (oral); the dose was increased progressively up to 2 mg/day or 4 mg/day for a total treatment period of 34 or 58 weeks, as previously described [22] (Fig. 1). Data from patients with microalbuminuria, defined as a UACR between 30.0 and 299.9 mg/g at baseline (i.e., prior to dotinurad administration) [26], were used in this post hoc analysis.

The inclusion and exclusion criteria for the phase 3 study have been previously published [22]. Briefly, patients with ≥ 7.0 mg/dL serum uric acid (in those with a history of gouty arthritis or gouty tophi); ≥ 8.0 mg/dL serum uric acid (in those with asymptomatic hyperuricemia diagnosed with or treated for diabetes mellitus, hypertension, and/or metabolic syndrome); or ≥ 9.0 mg/dL serum uric acid (in patients without these complications) during the run-in period were included. Patients with active gouty arthritis within the 2 weeks before enrollment and patients with uric acid overproduction were excluded. Urinary alkalinizing agents could be prescribed in the following scenarios: in patients with a history of urinary tract stones; in patients with a urinary pH < 6.0 on clinical examination; or at the physician’s discretion.

### Study endpoints

In the main clinical trial, the endpoint was the percentage reduction in serum uric acid levels [22]; in this post hoc analysis the endpoint was the change in UACR from baseline at weeks 34 and 58 after dotinurad administration. Subgroup analyses by patient background and clinical characteristics were performed.

### Statistical analysis

Summary statistics and 95% confidence intervals (CI) were calculated and a mixed effects model was used to test for pre- and post-dose changes; α was set at 0.05. Pre- and post-dose UACR were log-transformed, and the geometric mean and 95% CI of the percentage change were calculated. Logarithmic values were then restored. Fixed effects were set as baseline values and time points of the UACR; patients were included as a variable effect (random effect) and the objective variable was the UACR value at each measurement point. Patient effects were modelled, including time course, and estimated using a linear mixed-effects model accounting for repeated measurements. Univariate and multivariate analyses were performed to correlate patient background and clinical parameters with changes of UACR. All statistical analyses were performed using SAS version 9.4 (SAS Institute; Cary, NC, USA).

## Results

### Patients

Data from 39 patients with microalbuminuria at baseline were extracted from the original phase 3 study and used in this post hoc analysis. The demographic and clinical characteristics of those patients are shown in Table 1. All were male, and the mean (standard deviation) age was 57.5 (9.6) years, body mass index (BMI) was 28.1 (4.0) kg/m^2^, and UACR was 64.7 (0.7) mg/g. Urinary alkalinizing agents were prescribed in 37 of 39 patients.

**Table 1:** Demographic and clinical characteristics (microalbuminuria population)

Characteristic		Patients (*N* = 39)
Age (years)	Mean (SD) [95% CI]	57.5 (9.6) [54.4, 60.7]
Body weight (kg)	Mean (SD) [95% CI]	81.7 (14.5) [77.1, 86.4]
BMI (kg/m^2^)	Mean (SD) [95% CI]	28.1 (4.0) [26.8, 29.4]
SBP (mmHg)	Mean (SD) [95% CI]	141.8 (14.4) [137.1, 146.5]
DBP (mmHg)	Mean (SD) [95% CI]	88.1 (11.5) [84.4, 91.9]
Serum uric acid (mg/dL)	Mean (SD) [95% CI]	8.9 (0.9) [8.6, 9.2]
eGFR (mL/min/1.73 m^2^)	Mean (SD) [95% CI]	67.2 (16.5) [61.8, 72.5]
UACR (mg/g)	Mean (SD) [95% CI]	64.7 (0.7) [53.0, 78.9]
HOMA-IR	Mean (SD) [95% CI]	2.6 (2.0) [0.3, 11.3]
HbA1c	Mean (SD) [95% CI]	6.0 (0.6) [5.0, 7.5]
Sex	Male	39 (100)
Medical history of hyperuricemia	No	16 (41.0)
	Yes	23 (59.0)
History of gouty arthritis	No	11 (28.2)
	Yes	28 (71.8)
Comorbidities	No	0 (0)
	Yes	39 (100)
Hypertension	No	6 (15.4)
	Yes	33 (84.6)
Diabetes mellitus	No	28 (71.8)
	Yes	11 (28.2)
CKD	No	36 (92.3)
	Yes	3 (7.7)
Cardiovascular disorders	No	36 (92.3)
	Yes	3 (7.7)
Concomitant medications	No	0 (0)
	Yes	39 (100)
ARBs	No	21 (53.8)
	Yes	18 (46.2)
Beta blockers	No	36 (92.3)
	Yes	3 (7.7)
ACE inhibitors	No	39 (100)
	Yes	0 (0)
SGLT2 inhibitors	No	39 (100)
	Yes	0 (0)
DPP-4 inhibitors	No	34 (87.2)
	Yes	5 (12.8)
GLP-1 agonists	No	39 (100)
	Yes	0 (0)
Statins	No	28 (71.8)
	Yes	11 (28.2)

Data are shown as *n* (%) unless otherwise indicated

*ACE* angiotensin-converting enzyme, *ARB* angiotensin II receptor blocker, *BMI* body mass index, *CI* confidence interval, *CKD* chronic kidney disease, *DBP* diastolic blood pressure, *DPP-4* dipeptidyl-peptidase 4, *eGFR* estimated glomerular filtration rate, *GLP-1* glucagon-like peptide-1, *HbA1c* glycated hemoglobin, *HOMA-IR* Homeostatic Model Assessment for Insulin Resistance, *SBP* systolic blood pressure, *SD* standard deviation, *SGLT2* sodium-glucose cotransporter-2, *UACR* urine albumin-to-creatinine ratio

For reference, demographic and clinical characteristics for the total phase 3 population are shown in Online Resource 1. Compared with the total phase 3 population, patients in the microalbuminuria population tended to have higher mean body weight (81.7 kg vs 76.8 kg), BMI (28.1 kg/m^2^ vs 26.4 kg/m^2^), systolic blood pressure (SBP; 141.8 mmHg vs 134.9 mmHg), and diastolic blood pressure (88.1 mmHg vs 84.3 mmHg).

### Efficacy

**Fig. 2:**
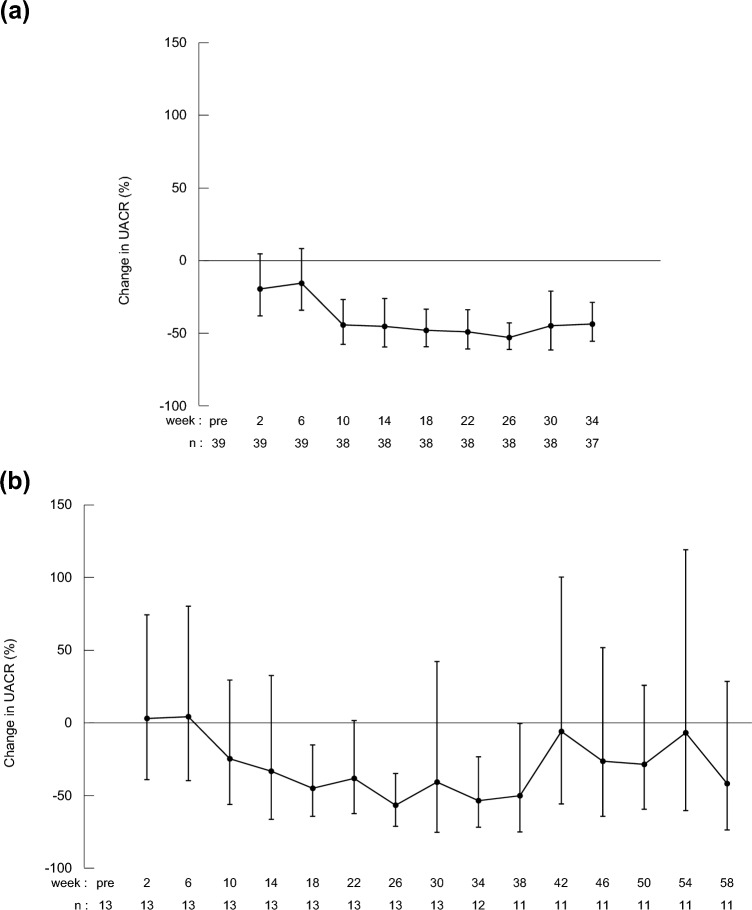
Temporal changes in UACR (microalbuminuria population). **(a)** shows combined data for the 34-week and 58-week group up to 34 weeks; **(b)** shows data for the 58-week group. Two patients discontinued before 34 weeks. A linear mixed-effects model was used to test for post-dose changes from pre-dose. P-values were < 0.05 for 2 to 58 weeks. *UACR* urine albumin-to-creatinine ratio

**Fig. 3:**
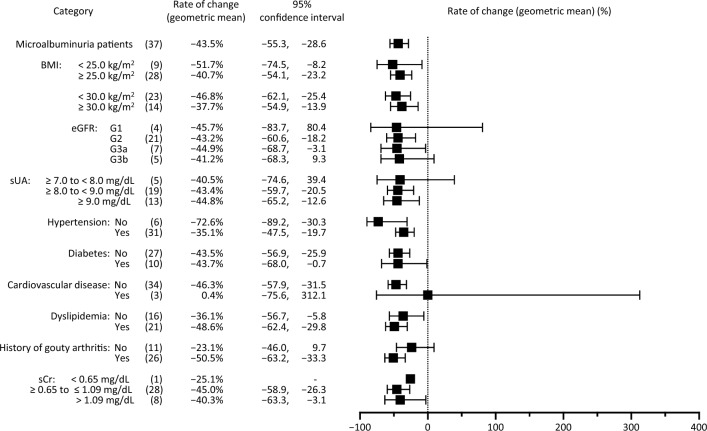
Subgroup analysis by baseline characteristics and rate of change in UACR at 34 weeks (microalbuminuria population). *BMI* body mass index, *eGFR* estimated glomerular filtration rate, *sCr* serum creatinine, *sUA* serum uric acid, *UACR* urine albumin-to-creatinine ratio

Among patients with microalbuminuria, the geometric mean of the change in UACR at Week 34 was −43.5% (95% CI −55.3%, −28.6%; linear mixed effects model, p < 0.05) (Fig. 2A and Table 2). At Week 58, the geometric mean of the change was −41.5% (95% CI −73.5%, 29.1%; linear mixed effects model, p < 0.05) (Fig. 2B**)**. At Week 34, 37.8% (*n* = 14) of patients had normal UACR (< 30.0 mg/g); 62.2% (*n* = 23) had UACR 30.0–299.9 mg/g; and 0% had UACR ≥ 300 mg/g. At Week 58, 36.4% (*n* = 4) of patients had normal UACR (< 30.0 mg/g); 54.5% (*n* = 6) had UACR 30.0–299.9 mg/g; and 9.1% (*n* = 1) had UACR ≥ 300 mg/g. (Table 2). For reference, similar data for the total phase 3 population are shown in Online Resources 2 and 3, including six patients with macroalbuminuria (UACR ≥ 300.0 mg/g) prior to dotinurad administration (Online Resource 3). Background characteristics of the 11 patients who received dotinurad through 58 weeks are shown in Online Resource 4. In subgroup analyses at Week 34, potential correlations between the rate of change in UACR and background parameters were not detected among patients with microalbuminuria (Fig. 3) or in the total population (Online Resource 5).

**Table 2:** UACR, change in UACR, and UACR range over time (microalbuminuria population)

Evaluation	*n*	UACR (mg/g)						UACR range		
		Actual measurement (geo mean)		Change		% change (geo mean)		0.0–29.9 mg/g	30.0–299.9 mg/g	≥ 300.0 mg/g
		Geo mean	95% CI	Mean	95% CI	Geo mean	95% CI	*n* (%)	*n* (%)	*n* (%)
Pre	39	64.7	53.0, 78.9	–	–	–	–	–	–	–
34 weeks	37	37.1	26.7, 51.6	− 23.9	− 37.7, − 10.0	− 43.5	− 55.3, − 28.6	14 (37.8)	23 (62.2)	0 (0.0)
58 weeks	11	37.9	13.6, 105.3	4.3	− 34.6, 43.1	− 41.5	− 73.5, 29.1	4 (36.4)	6 (54.5)	1 (9.1)

P-value for UACR percent change (geo mean) was p < 0.05 at Weeks 34 and 58

*CI* confidence interval, *geo* geometric, *UACR* urine albumin-to-creatinine ratio

Table 3 shows the correlation between patient clinical parameters before dotinurad administration and the rate of change in UACR after dotinurad administration. At Week 34, there was a significant correlation between SBP in both the univariate and multivariate analyses. At Week 58, there was a significant correlation with SBP, Homeostatic Model Assessment for Insulin Resistance (HOMA-IR), and high-density lipoprotein cholesterol in the univariate analysis and SBP and HOMA-IR in the multivariate analysis. When evaluating the total phase 3 population, sex was the only factor that correlated with rate of change in UACR in both univariate and multivariate analyses (Online Resource 6). Regarding temporal changes in SBP, there was a slight decrease compared with baseline from Week 2 in the 34-week group, but no clear changes were observed; in the 58-week group, the number of patients was small (*n* = 11) and variability was high (Online Resource 7). Scatter plots of the relationships between change in UACR and either change in serum uric acid from baseline or change in SBP from baseline are shown in Online Resources 8 and 9, respectively. Regarding the relationship with change in serum uric acid, R^2^ was 0.0053 for the 34-week group and 0.0068 for the 58-week group (Online Resource 8); respective R^2^ values for the relationship with change in SBP were 0.0031 and 0.004 (Online Resource 9), suggesting no clear correlation between change in UACR and either change in serum uric acid or change in SBP.

**Table 3:** Correlation between patient clinical characteristics and rate of change in UACR (microalbuminuria population)

Evaluation period	Item (data)	UACR (corrected) (mg/g) (rate of change)						
		Univariate			Multivariate			
		R^2^	Regression coefficient	p-value	Regression coefficient	Standard partial regression coefficient	VIF	p-value
34 weeks	sUA change (mg/dL)	0.0056	− 2.8843	0.6653	–	–	–	–
	sUA rate of change (%)	0.0042	− 0.2506	0.7071	–	–	–	–
	Achieved sUA ≤ 6 mg/dL	0.0043	7.4906	0.7049	–	–	–	–
	sUA level before administration (mg/dL)	0.0001	− 0.5253	0.9459	–	–	–	–
	Age (years)	0.0007	0.1155	0.8786	–	–	–	–
	Weight (kg)	0.0105	0.2962	0.5467	–	–	–	–
	BMI (kg/m^2^)	0.0021	0.4800	0.7889	–	–	–	–
	SBP (mmHg)	0.2069	1.3327	0.0047	1.3402	0.4574	1.0000	0.0053
	DBP (mmHg)	0.0295	0.6233	0.3093	–	–	–	–
	HbA1c (%)	0.0034	3.9555	0.7315	–	–	–	–
	HOMA-IR	0.0048	− 1.4201	0.6828	–	–	–	–
	eGFR (mL/min/1.73m^2^)	0.0001	− 0.0256	0.9530	–	–	–	–
	LDL-C (mg/dL)	0.0057	− 0.1428	0.6579	–	–	–	–
	HDL-C (mg/dL)	0.0011	− 0.0954	0.8426	–	–	–	–
	TG (mg/dL)	0.0005	− 0.0065	0.8930	–	–	–	–
58 weeks	sUA change (mg/dL)	0.0051	− 2.9473	0.8343	–	–	–	–
	sUA rate of change (%)	0.0089	− 0.4135	0.7824	–	–	–	–
	Achieved sUA ≤ 6 mg/dL	0.0705	47.3702	0.4300	–	–	–	–
	sUA level before administration (mg/dL)	0.0078	− 6.2923	0.7962	––	–	–	–
	Age (years)	0.3288	3.4273	0.0652	–	–	–	–
	Weight (kg)	0.3374	− 2.4176	0.0610	–	–	–	–
	BMI (kg/m^2^)	0.3482	− 11.8252	0.0560	–	–	–	–
	SBP (mmHg)	0.4289	3.1110	0.0287	2.4666	0.5193	1.0523	0.0157
	DBP (mmHg)	0.0096	0.6037	0.7748	–	–	–	–
	HbA1c (%)	0.0093	10.0398	0.7784	–	–	–	–
	HOMA-IR	0.5244	− 49.1420	0.0117	− 41.2842	− 0.6084	1.0523	0.0072
	eGFR (mL/min/1.73m^2^)	0.0018	− 0.2605	0.9027	–	–	–	–
	LDL-C (mg/dL)	0.0289	− 0.6133	0.6173	–	–	–	–
	HDL-C (mg/dL)	0.4233	2.6920	0.0302	–	–	–	–
	TG (mg/dL)	0.1474	− 0.2060	0.2437	–	–	–	–

The data used for the items sUA change, sUA change rate, and achievement of sUA ≤ 6 mg/dL were calculated using the time of evaluation; other items were calculated using pre-dose data

For the multivariate analysis, variable selection was performed using the variable increase/decrease method and the optimal model was selected. Outliers in the rate of change for urinary albumin/creatinine ratio (corrected value) (mg/g creatinine) were excluded

*BMI* body mass index, *DBP* diastolic blood pressure, *eGFR* estimated glomerular infiltration rate, *HbA1c* glycated hemoglobin, *HDL-C* high density lipoprotein cholesterol, *HOMA-IR* Homeostatic Model Assessment for Insulin Resistance, *LDL-C* low density lipoprotein cholesterol, *SBP* systolic blood pressure, *sUA* serum uric acid, *TG* triglyceride, *UACR* urine albumin-to-creatinine ratio, *VIF* variance inflation factor

## Discussion

Following dotinurad initiation, a marked decrease in UACR was observed in patients with microalbuminuria, reaching a plateau by 14–18 weeks. Considering the titration of the dotinurad dose, which was increased from 0.5 mg/day at study initiation to 1 mg/day after 2 weeks and 2 mg/day after 6 weeks, it appears that the decrease in UACR plateaued around the time the maximum dotinurad dose was administered. Although the mechanism by which dotinurad lowers urinary albumin is currently unknown, this suggests that dotinurad was responsible for the decrease in UACR and may be because of the inhibitory effect of URAT1.

The trend towards decreased UACR tended to be greater among patients with microalbuminuria compared with the total phase 3 population; however, this should be interpreted with caution because of the small number of patients with microalbuminuria and the floor effect observed in the total phase 3 population. The results of observational studies [27–29] also suggest that dotinurad administration improves urinary albumin levels or estimated glomerular filtration rate, consistent with the findings of the current study. Nevertheless, the number of patients with macroalbuminuria in the total phase 3 population was very small, so caution is warranted in interpreting the effects of dotinurad on albuminuria (Online Resource 3).

In addition, most patients in whom this analysis was conducted were considered to have conditions of metabolic syndrome such as hypertension and obesity. In other words, many patients were presumed to have metabolic syndrome. Benzbromarone—also a uricosuric agent—is suggested to have insulin resistance-improving effects [30]. Therefore, it is plausible that dotinurad may also lower urinary albumin by improving insulin resistance in hyperinsulinemic patients.

There were no significant correlations between patient background and the UACR-lowering effects of dotinurad. Of particular interest, subgroup analyses of all patients revealed that dotinurad had an albumin-lowering effect regardless of the presence of diabetes. The evidence that dotinurad lowers urinary albumin in this population is of interest, given the possibility that dotinurad may have a urinary albumin-lowering effect in patients with microalbuminuria and hyperuricemia. Urinary albumin testing in Japan is only accepted for reimbursement in diabetes mellitus or early diabetic nephropathy. In this study, only 11 (28.2%) patients had diabetes. All were male, with a mean age of 57.5 years and a mean BMI of 28.1 kg/m^2^; thus, as noted, some may have had concomitant metabolic syndrome (34-week group). Metabolic syndrome has been reported to be a factor strongly correlated with urinary albumin, and albuminuria may be missed in many cases in hyperuricemic patients with metabolic syndrome [31]. The development of new drugs with renoprotective effects is highly anticipated, as such drugs may be of benefit in our aging society.

SBP was the only patient clinical parameter that correlated with a decrease in UACR. While there may have been a tendency for a reduction in SBP following dotinurad initiation in the current study, no clear trends were observed, and R^2^ values suggested no clear correlation between change in SBP and change in UACR in either the 34- or 58-week groups. In patients with hypertension in the pooled analysis of phase 2 and phase 3 trials, similar SBP-lowering effects were observed at 2 weeks in the dotinurad 1 mg and placebo groups, suggesting that dotinurad was not responsible for these effects [32]. Furthermore, it is unlikely that there is a direct relationship between the URAT1 inhibitory effect of dotinurad and blood pressure, given that dotinurad effectively lowers urinary albumin in patients with hypertension [32]. Blood pressure and renal damage are interrelated, and the higher the blood pressure, the earlier the progression of renal damage [33–35].

There are three main associations between hyperuricemia and renal damage, including uric acid crystals, increased blood pressure via the renin–angiotensin system, and increased concentrations of intracellular uric acid [36, 37]. Uric acid crystals and the renin–angiotensin system are thought to be involved in the mechanism of uric acid-lowering drugs. Similar mechanisms may ameliorate renal impairment, thus decreasing albuminuria. When considering the association between increased intracellular concentrations of uric acid and kidney damage, dotinurad specifically inhibits tubular URAT1, which may reduce levels of intracellular uric acid to a greater extent than other uricosuric drugs. Furthermore, uricosuric drugs, such as probenecid, block the uric acid stimulation of nicotinamide adenine dinucleotide phosphate oxidase and its subsequent translocation to the mitochondria [38]. It is possible that dotinurad acts through a similar mechanism to decrease intracellular concentrations of uric acid and reduce oxidative stress. While the relationship between serum uric acid levels and the risk of poor renal outcomes is not fully understood, it has been reported to differ depending on sex and age [39–41].

This study has some limitations. First, clinical trial data differ from real-world data; thus, the findings should be evaluated in real-world settings. Second, the findings were not compared with a placebo group. Third, the study comprised only male patients, and the findings cannot be generalized to females. Fourth, some patients took concomitant angiotensin II receptor blockers and/or sodium-glucose cotransporter-2 inhibitors, which may have affected the urinary albumin-lowering effect. Fifth, analysis of change in UACR was performed only using a linear mixed-effects model, whereas changes in UACR may have been non-linear. Sixth, it cannot be denied that the decrease in UACR following dotinurad administration is due to a temporal effect. Finally, this post hoc analysis was conducted using data from a small number of patients, particularly after Week 38; therefore, the findings should be interpreted with caution.

In conclusion, our study found that there was a trend toward a decrease in the UACR following dotinurad administration, although, the mechanism for this remains unknown. RCTs with appropriate control groups are needed to evaluate the effect of dotinurad on urinary albumin.

## Supplementary Information

Below is the link to the electronic supplementary material.

Supplementary file1 (DOCX 582 KB)

## Data Availability

Data may be made available on request but are not publicly available for patient confidentiality reasons.
