# Supplementary material for: Changes in urinary albumin levels with dotinurad oral administration in hyperuricemic patients with microalbuminuria: a post hoc analysis
Source: Clin Exp Nephrol. 2025 Aug 21;29(10):1384–93. doi: 10.1007/s10157-025-02750-4 (PMC12464093; doi:10.1007/s10157-025-02750-4)
Supplement: Supplementary file 1 — Supplementary file1 (DOCX 582 KB) [file 10157_2025_2750_MOESM1_ESM.docx]

**Supplemental Material**

**Changes in urinary albumin levels with dotinurad oral administration in hyperuricemic patients with microalbuminuria: a *post hoc* analysis**

***Clinical and Experimental Nephrology***

Toshinari Takahashi, Takanobu Beppu, Tatsuo Hosoya, Naoto Yokota

**Corresponding author:**

Takanobu Beppu

Medical Affairs Department, Fuji Yakuhin Co., Ltd, 9F Kanda Square Building, 2-2-1 Kandanishiki-cho, Chiyoda City, Tokyo 101-8189, Japan

Email: beppu@fujiyakuhin.co.jp

Phone: +81-3-6811-7834

Fax: +81-3-6811-7864

**Online Resource 1** Demographic and clinical characteristics (total phase 3 population)

| Characteristic | Patients  (*N* = 326) |
| --- | --- |
| Age (years) | 53.9 (10.5) [52.8, 55.1] |
| Body weight (kg) | 76.8 (12.4) [75.4, 78.1] |
| BMI (kg/m^2^) | 26.4 (3.8) [26.0, 26.8] |
| SBP (mmHg) | 134.9 (14.9) [133.2, 136.5] |
| DBP (mmHg) | 84.3 (11.1) [83.1, 85.5] |
| Serum uric acid (mg/dL) | 8.8 (1.1) [8.7, 8.9] |
| eGFR (mL/min/1.73 m^2^) | 69.6 (13.2) [68.1, 71.0] |
| UACR (mg/g) | 7.80 (1.94) [6.80, 8.93] |
| Serum creatinine (mg/dL) | 0.9 (0.2) [0.9, 0.9] |
| Urea nitrogen (mg/dL) | 14.0 (3.8) [13.6, 14.4] |
| LDL-C (mg/dL) | 121.9 (31.6) [118.5, 125.4] |
| HDL-C (mg/dL) | 57.8 (14.0) [56.3, 59.3] |
| TG (mg/dL) | 161.5 (112.2) [149.2, 173.7] |
| HOMA-IR | 1.8 (1.6) [1.6, 2.0] |
| HbA1c (%) | 5.8 (0.5) [5.7, 5.8] |

Data are shown as mean (SD) [95% CI].

*BMI* body mass index, *CI* confidence interval, *DBP* diastolic blood pressure, *eGFR* estimated glomerular filtration rate, *HbA1c* glycated hemoglobin, *HDL-C* high density lipoprotein cholesterol, *HOMA-IR* Homeostatic Model Assessment for Insulin Resistance, *LDL-C* low density lipoprotein cholesterol, *SBP* systolic blood pressure, *SD* standard deviation, *TG* triglyceride, *UACR* urine albumin-to-creatinine ratio.

**Online Resource 2** Clinical characteristics (total phase 3 population, *N* = 326)

| Characteristics |  | Patients, *n* (%) |
| --- | --- | --- |
| Sex | Male | 324 (99.4) |
|  | Female | 2 (0.6) |
| Medical history of hyperuricemia | No | 180 (55.2) |
|  | Yes | 146 (44.8) |
| History of gouty arthritis | No | 55 (16.9) |
|  | Yes | 271 (83.1) |
| History of alcohol use | No | 156 (47.9) |
|  | Yes | 170 (52.1) |
| Type of uricemia |  |  |
| Underexcretion type | No | 47 (14.4) |
|  | Yes | 279 (85.6) |
| Mixed type/normal type | No | 279 (85.6) |
|  | Yes | 47 (14.4) |
| History of urinary tract stone disease | No | 291 (89.3) |
|  | Yes | 35 (10.7) |
| Comorbidities | No | 42 (12.9) |
|  | Yes | 284 (87.1) |
| Hypertension | No | 167 (51.2) |
|  | Yes | 159 (48.8) |
| Diabetes mellitus | No | 290 (89.0) |
|  | Yes | 36 (11.0) |
| CKD | No | 317 (97.2) |
|  | Yes | 9 (2.8) |
| Cardiovascular disorders | No | 310 (95.1) |
|  | Yes | 16 (4.9) |
| Dyslipidemia | No | 193 (59.2) |
|  | Yes | 133 (40.8) |
| Atrial fibrillation | No | 319 (97.9) |
|  | Yes | 7 (2.1) |
| Concomitant medications | No | 8 (2.5) |
|  | Yes | 318 (97.5) |
| ARB | No | 263 (80.7) |
|  | Yes | 63 (19.3) |
| CCB | No | 287 (88.0) |
|  | Yes | 39 (12.0) |
| SGLT2 inhibitor | No | 324 (99.4) |
|  | Yes | 2 (0.6) |
| GLP-1 agonist | No | 326 (100) |
|  | Yes | 0 (0) |
| Statin | No | 283 (86.8) |
|  | Yes | 43 (13.2) |
| DPP-4 | No | 310 (95.1) |
|  | Yes | 16 (4.9) |
| Thiazide diuretic | No | 319 (97.9) |
|  | Yes | 7 (2.1) |
| Loop diuretic | No | 323 (99.1) |
|  | Yes | 3 (0.9) |
| Beta blockers | No | 309 (94.8) |
|  | Yes | 17 (5.2) |
| ACE inhibitor | No | 324 (99.4) |
|  | Yes | 2 (0.6) |
| Losartan | No | 323 (99.1) |
|  | Yes | 3 (0.9) |

*ACE* angiotensin-converting enzyme, *ARB* angiotensin II receptor blocker, *CCB* calcium channel blocker, *CKD* chronic kidney disease, *DPP-4* dipeptidyl-peptidase 4, *GLP-1* glucagon-like peptide-1, *SGLT2* sodium-glucose cotransporter-2.

**Online Resource 3** UACR, change in UACR, and UACR range over time (total phase 3 population, *N* = 326)

| Evaluation (weeks) | UACR range | *n* | UACR (mg/g) | | | | | |
| --- | --- | --- | --- | --- | --- | --- | --- | --- |
|  |  |  | Actual measurement (Geo mean) | | Change | | % change (Geo mean) | |
|  |  |  | Geo mean | 95% CI | Mean | 95% CI | Geo mean | 95% CI |
| Pre | 30.0–299.9 mg/g | 39 | 64.7 | 53.0, 78.9 | - | - | - | - |
|  | ≥ 300.0 mg/g | 6 | 733.9 | 349.7, 1540.1 | - | - | - | - |
|  | Total population | 326 | 7.8 | 6.8, 8.9 | - | - | - | - |
| 2 | 30.0–299.9 mg/g | 39 | 52.1 | 36.9, 73.7 | 0.4 | −13.5, 14.3 | −19.4 | −37.9, 4.7 |
|  | ≥ 300.0 mg/g | 6 | 543.1 | 207.2, 1423.3 | −139.0 | −346.8, 68.8 | −26.0 | −52.6, 15.5 |
|  | Total population | 325 | 7.8 | 6.8, 9.0 | −0.8 | −4.7, 3.1 | −0.3 | −7.4, 7.4 |
| 6 | 30.0–299.9 mg/g | 39 | 54.7 | 40.7, 73.4 | −3.7 | −21.1, 13.6 | −15.5 | −34.0, 8.3 |
|  | ≥ 300.0 mg/g | 6 | 569.8 | 274.9, 1180.9 | −203.6 | −457.5, 50.4 | −22.4 | −48.8, 17.7 |
|  | Total population | 322 | 7.4 | 6.4, 8.5 | −3.0 | −8.3, 2.3 | −5.4 | −11.9, 1.6 |
| 10 | 30.0–299.9 mg/g | 38 | 36.2 | 25.7, 51.2 | −18.8 | −39.9, 2.2 | −44.2 | −57.5, −26.7 |
|  | ≥ 300.0 mg/g | 6 | 424.4 | 113.8, 1582.2 | −192.3 | −612.1, 227.5 | −42.2 | −79.6, 63.9 |
|  | Total population | 314 | 6.5 | 5.6, 7.4 | −5.7 | −12.6, 1.3 | −16.8 | −22.9, −10.3 |
| 14 | 30.0–299.9 mg/g | 38 | 35.6 | 24.3, 52.2 | −15.7 | −35.0, 3.6 | −45.1 | −59.3, −26.0 |
|  | ≥ 300.0 mg/g | 6 | 592.8 | 232.0, 1514.3 | −95.0 | −362.5, 172.6 | −19.2 | −53.3, 39.7 |
|  | Total population | 312 | 5.9 | 5.1, 6.8 | −4.6 | −9.1, 0.0 | −24.5 | −30.2, −18.3 |
| 18 | 30.0–299.9 mg/g | 38 | 33.9 | 24.9, 46.1 | −25.7 | −46.0, −5.3 | −47.8 | −59.2, −33.3 |
|  | ≥ 300.0 mg/g | 6 | 427.1 | 129.5, 1408.1 | −208.1 | −569.6, 153.4 | −41.8 | −75.9, 40.5 |
|  | Total population | 312 | 6.0 | 5.2, 6.8 | −7.8 | −14.2, −1.5 | −23.1 | −28.6, −17.2 |
| 22 | 30.0–299.9 mg/g | 38 | 33.1 | 24.6, 44.7 | −28.8 | −49.2, −8.4 | −48.9 | −60.6, −33.7 |
|  | ≥ 300.0 mg/g | 6 | 490.9 | 207.0, 1164.3 | −271.5 | −676.5, 133.6 | −33.1 | −68.3, 41.2 |
|  | Total population | 307 | 6.0 | 5.3, 6.9 | −9.3 | −16.8, −1.9 | −22.9 | −28.4, −17.0 |
| 26 | 30.0–299.9 mg/g | 38 | 30.6 | 23.3, 40.3 | −38.2 | −52.6, −23.8 | −52.8 | −61.0, −42.8 |
|  | ≥ 300.0 mg/g | 6 | 469.3 | 146.7, 1501.7 | −208.2 | −470.6, 54.3 | −36.0 | −69.0, 32.1 |
|  | Total population | 305 | 5.8 | 5.1, 6.7 | −9.4 | −14.8, −4.1 | −25.9 | −31.0, −20.4 |
| 30 | 30.0–299.9 mg/g | 38 | 35.9 | 24.1, 53.4 | 12.5 | −70.1, 95.1 | −44.7 | −61.4, −20.9 |
|  | ≥ 300.0 mg/g | 6 | 710.5 | 275.7, 1831.5 | 29.4 | −425.5, 484.4 | −3.2 | −54.4, 105.6 |
|  | Total population | 302 | 6.3 | 5.4, 7.3 | 2.9 | −9.1, 14.8 | −20.6 | −27.2, −13.4 |
| 34 | 30.0–299.9 mg/g | 37 | 37.1 | 26.7, 51.6 | −23.9 | −37.7, −10.0 | −43.5 | −55.3, −28.6 |
|  | ≥ 300.0 mg/g | 6 | 794.8 | 288.2, 2191.8 | 194.3 | -387.4, 776.1 | 8.3 | −55.6, 164.2 |
|  | Total population | 299 | 6.1 | 5.2, 7.1 | 0.9 | −8.1, 9.9 | −23.1 | −28.7, −17.0 |
| 38 | 30.0–299.9 mg/g | 11 | 32.5 | 12.8, 82.7 | −6.8 | −59.3, 45.7 | −49.9 | −74.9, 0.1 |
|  | ≥ 300.0 mg/g | 1 | 317.3 | - | −24.4 | - | −7.1 | - |
|  | Total population | 107 | 5.8 | 4.6, 7.3 | −1.2 | −6.0, 3.6 | −21.4 | −31.8, −9.4 |
| 42 | 30.0–299.9 mg/g | 11 | 61.3 | 23.8, 158.0 | 42.8 | −25.0, 110.7 | −5.5 | −55.6, 101.2 |
|  | ≥ 300.0 mg/g | 1 | 506.4 | - | 164.7 | - | 48.2 | - |
|  | Total population | 106 | 6.4 | 5.0, 8.4 | 6.5 | −0.8, 13.7 | −12.6 | −24.5, 1.0 |
| 46 | 30.0–299.9 mg/g | 11 | 47.9 | 19.9, 115.3 | 14.4 | −60.4, 89.2 | −26.1 | −64.2, 52.5 |
|  | ≥ 300.0 mg/g | 1 | 378.8 | - | 37.1 | - | 10.86 | - |
|  | Total population | 105 | 6.2 | 4.7, 8.0 | 3.7 | −3.5, 10.9 | −17.3 | −28.6, −4.2 |
| 50 | 30.0–299.9 mg/g | 11 | 46.5 | 19.5, 110.9 | 8.2 | −29.3, 45.6 | −28.2 | −59.3, 26.4 |
|  | ≥ 300.0 mg/g | 1 | 546.2 | - | 204.5 | - | 59.8 | - |
|  | Total population | 105 | 6.7 | 5.2, 8.7 | 4.7 | −0.8, 10.2 | −7.6 | −19.8, 6.5 |
| 54 | 30.0–299.9 mg/g | 11 | 60.7 | 21.7, 169.8 | 51.8 | −32.1, 135.8 | −6.4 | −60.2, 120.1 |
|  | ≥ 300.0 mg/g | 1 | 478.5 | - | 136.8 | - | 40.0 | - |
|  | Total population | 104 | 6.8 | 5.1, 9.1 | 9.4 | 0.5, 18.4 | −6.7 | −20.4, 9.5 |
| 58 | 30.0–299.9 mg/g | 11 | 37.9 | 13.6, 105.3 | 4.3 | −34.6, 43.1 | −41.5 | −73.5, 29.1 |
|  | ≥ 300.0 mg/g | 1 | 426.5 | - | 84.8 | - | 24.8 | - |
|  | Total population | 105 | 5.3 | 4.1, 6.8 | 1.5 | −2.9, 5.9 | −27.7 | −37.6, −16.3 |
| Final | 30.0–299.9 mg/g | 39 | 38.5 | 26.9, 55.0 | −14.2 | −29.4, 1.0 | −40.5 | −54.7, −22.0 |
|  | ≥ 300.0 mg/g | 6 | 817.3 | 304.3, 2194.8 | 205.3 | −372.8, 783.3 | 11.4 | −54.4, 172.2 |
|  | Total population | 326 | 6.2 | 5.4, 7.2 | 2.5 | −5.8, 10.8 | −20.1 | −26.0, −13.6 |

*CI* confidence interval, *geo* geometric, *UACR* urine albumin-to-creatinine ratio.

**Online Resource 4** Background characteristics of patients who received dotinurad through 58 weeks (microalbuminuria population; *N =* 11)

| Patient | Age (y) | Final dose (mg) | BMI (kg/m^2^) | sUA (mg/dL) | Baseline eGFR (mL/min/1.73m^2^) | UACR (mg/g) | HOMA-IR | SBP (mmHg) | DBP (mmHg) | Serum creatinine (mg/dL) | Comorbidities |
| --- | --- | --- | --- | --- | --- | --- | --- | --- | --- | --- | --- |
| 1 | 43 | 2 | 30.2 | 8.6 | 71 | 135.9 | 3.1 | 146 | 102.0 | 0.93 | Hypertension, T2DM,atrial fibrillation, thoracic disc herniation |
| 2 | 70 | 2 | 24.9 | 8.7 | 50 | 147.9 | 1.0 | 156 | 80.0 | 1.13 | Hypertension, diabetes mellitus, dyslipidemia, angina pectoris, knee pain |
| 3 | 64 | 2 | 26.7 | 8.3 | 70 | 46.8 | 3.4 | 157 | 104.0 | 0.85 | Hypertension, T2DM |
| 4 | 65 | 4 | 23.5 | 9.5 | 73 | 46.7 | 2.6 | 138 | 66.0 | 0.81 | Dyslipidemia |
| 5 | 69 | 4 | 23.0 | 9.8 | 64 | 30.2 | 2.2 | 126 | 85.0 | 0.90 | Allergic rhinitis, tooth decay |
| 6 | 46 | 2 | 31.1 | 8.8 | 65 | 37.8 | 2.5 | 137 | 89.0 | 0.99 | Fatty liver, hepatic cyst, renal cyst, diverticulum coli |
| 7 | 67 | 2 | 24.0 | 11.6 | 39 | 46.0 | 1.0 | 136 | 80.0 | 1.40 | Hypertension, renal impairment, renal cyst, prostatic hypertrophy, lumbago, allergic rhinitis, allergic conjunctivitis, ptosis, dentition |
| 8 | 73 | 2 | 24.4 | 7.7 | 82 | 199.6 | 0.4 | 133 | 85.0 | 0.71 | Hypertension, reflux esophagitis, bilateral knee osteoarthritis, atherosclerosis, chronic atrophic gastritis, periarthritis of both shoulders |
| 9 | 48 | 2 | 32.4 | 9.5 | 59 | 54.8 | 2.4 | 142 | 100.0 | 1.06 | Hypertension, dyslipidemia, fatty liver, gallstones, gall bladder polyps, allergic conjunctivitis |
| 10 | 70 | 2 | 21.5 | 9.3 | 61 | 48.0 | 0.3 | 179 | 100.0 | 0.94 | Hypertension, atrial fibrillation, eczema, nail dryness |
| 11 | 43 | 2 | 27.5 | 8.8 | 65 | 70.2 | 1.9 | 131 | 92.0 | 1.00 | Hypertension, dyslipidemia, fatty liver, hepatic cysts, sleep apnea syndrome, gallbladder polyp, hay fever |

*BMI* body mass index, *DBP* diastolic blood pressure, *eGFR* estimated glomerular infiltration rate, *HOMA-IR* Homeostatic Model Assessment for Insulin Resistance, *SBP* systolic blood pressure, *sUA* serum uric acid, *T2DM* type 2 diabetes mellitus, *UACR* urine albumin-to-creatinine ratio.

**Online Resource 5** Subgroup analysis by baseline characteristics and rate of change in UACR (total phase 3 population, *N* = 326; results from 299 cases at 34 weeks)

**

*BMI* body mass index, *eGFR* estimated glomerular filtration rate, *sCr* serum creatinine, *sUA* serum uric acid, *UACR* urine albumin-to-creatinine ratio.

**Online Resource 6** Correlation between patient clinical characteristics and rate of change in UACR (total phase 3 population, *N* = 326)

| Evaluation period | Item (data) | UACR (corrected value) (mg/g)  (rate of change) | | | | | | |
| --- | --- | --- | --- | --- | --- | --- | --- | --- |
|  |  | Univariate | | | Multivariate | | | |
|  |  | R^2^ | Regression coefficient | p-value | Regression coefficient | Standard partial regression coefficient | VIF | p-value |
| 34 weeks | sUA change (mg/dL) | 0.0020 | −2.7586 | 0.4405 | - | - | - | - |
|  | sUA rate of change (%) | 0.0001 | −0.0488 | 0.8985 | - | - | - | - |
|  | Achieved sUA below 6 mg/dL | 0.0082 | 21.6819 | 0.1198 | - | - | - | - |
|  | sUA level before administration (mg/dL) | 0.0032 | 3.6525 | 0.3333 | - | - | - | - |
|  | Age (years) | 0.0006 | 0.1651 | 0.6763 | - | - | - | - |
|  | Sex | 0.0027 | 45.5285 | 0.3677 | - | - | - | - |
|  | Weight (kg) | 0.0016 | 0.2257 | 0.4906 | - | - | - | - |
|  | BMI (kg/m^2^) | 0.0040 | 1.1810 | 0.2743 | - | - | - | - |
|  | SBP (mmHg) | 0.0003 | −0.0763 | 0.7837 | - | - | - | - |
|  | DBP (mmHg) | 0.0029 | −0.3381 | 0.3518 | - | - | - | - |
|  | HbA1c (%) | 0.0009 | 4.1844 | 0.6096 | - | - | - | - |
|  | HOMA-IR | 0.0031 | 2.4419 | 0.3350 | - | - | - | - |
|  | eGFR (mL/min/1.73 m^2^) | 0.0007 | −0.1449 | 0.6449 | - | - | - | - |
|  | LDL-C (mg/dL) | 0.0103 | −0.2275 | 0.0805 | −0.2263 | −0.1009 | 1.0000 | 0.0833 |
|  | HDL-C (mg/dL) | 0.0023 | −0.2392 | 0.4084 | - | - | - | - |
|  | TG (mg/dL) | 0.0038 | 0.0390 | 0.2883 | - | - | - | - |
| 58 weeks | sUA change (mg/dL) | 0.0162 | −8.3159 | 0.1962 | - | - | - | - |
|  | sUA rate of change (%) | 0.0182 | −0.9742 | 0.1700 | −1.0769 | −0.1492 | 1.0025 | 0.1160 |
|  | Achieved sUA below 6 mg/dL | 0.0061 | 23.9766 | 0.4302 | - | - | - | - |
|  | sUA level before administration (mg/dL) | 0.0024 | 3.6625 | 0.6203 | - | - | - | - |
|  | Age (years) | 0.0072 | 0.6617 | 0.3887 | - | - | - | - |
|  | Sex | 0.0770 | 233.6034 | 0.0042 | 239.8740 | 0.2849 | 1.0025 | 0.0031 |
|  | Weight (kg) | 0.0056 | −0.4895 | 0.4488 | - | - | - | - |
|  | BMI (kg/m^2^) | 0.0026 | 1.2005 | 0.6065 | - | - | - | - |
|  | SBP (mmHg) | 0.0000 | −0.0267 | 0.9608 | - | - | - | - |
|  | DBP (mmHg) | 0.0009 | −0.2431 | 0.7663 | - | - | - | - |
|  | HbA1c (%) | 0.0049 | −11.2756 | 0.4763 | - | - | - | - |
|  | HOMA-IR | 0.0179 | −8.0938 | 0.1742 | - | - | - | - |
|  | eGFR (mL/min/1.73 m^2^) | 0.0004 | −0.1273 | 0.8457 | - | - | - | - |
|  | LDL-C (mg/dL) | 0.0023 | −0.1114 | 0.6260 | - | - | - | - |
|  | HDL-C (mg/dL) | 0.0012 | 0.2000 | 0.7213 | - | - | - | - |
|  | TG (mg/dL) | 0.0014 | −0.0331 | 0.7021 | - | - | - | - |

The data used for the items sUA change, sUA change rate, and achievement of sUA ≤ 6 mg/dL were calculated using the time of evaluation, other items were calculated using pre-dose data.

For the multivariate analysis, variable selection was performed using the variable increase/decrease method and the optimal model was selected. Outliers in the rate of change for urinary albumin/creatinine ratio (corrected value) (mg/g creatinine) were excluded.

*BMI* body mass index, *DBP* diastolic blood pressure, *eGFR* estimated glomerular infiltration rate, *HbA1c* glycated hemoglobin, *HDL-C* high density lipoprotein cholesterol, *HOMA-IR* Homeostatic Model Assessment for Insulin Resistance, *LDL-C* low density lipoprotein cholesterol, *SBP* systolic blood pressure, *sUA* serum uric acid, *TG* triglyceride, *UACR* urine albumin-to-creatinine ratio, *VIF* variance inflation factor.

**Online Resource 7** Temporal changes in systolic blood pressure (microalbuminuria population)

(A) combined data for the 34-week and 58-week group up to 34 weeks; (B) data for the 58-week group. Two patients discontinued before 34 weeks.

**Online Resource 8** Scatter plots of the relationship between change in UACR and change in sUA from baseline (microalbuminuria population)

(A) 34 weeks, *N* = 36; (B) 58 weeks, *N* = 11. sUA was missing for one case at week 34.

*sUA* serum uric acid, *UACR* urine albumin-to-creatinine ratio.

**Online Resource 9** Scatter plots of the relationship between change in UACR and change in systolic blood pressure from baseline (microalbuminuria population)

 (A) 34 weeks, *N* = 37; (B) 58 weeks, *N* = 11.

*UACR* urine albumin-to-creatinine ratio.
